# Interior Point-Driven Throughput Maximization for TS-SWIPT Multi-Hop DF Relays: A Log Barrier Approach

**DOI:** 10.3390/s25185901

**Published:** 2025-09-21

**Authors:** Yang Yu, Xiaoqing Tang, Guihui Xie

**Affiliations:** 1 Electronic Information School, Hubei Three Gorges Polytechnic, Yichang 443199, China; 2 School of Artificial Intelligence, Hubei University, Wuhan 430062, China; 2010202120076@whu.edu.cn; 3 School of Automation, China University of Geosciences (Wuhan), Wuhan 430074, China; xieguihui@cug.edu.cn

**Keywords:** decode and forward, interior point method, log barrier method, multi-hop relay, simultaneous wireless information and power transfer, time-switching

## Abstract

This paper investigates a simultaneous wireless information and power transfer (SWIPT) decode-and-forward (DF) relay network, where a source node transmits data to a destination node through the assistance of multi-hop passive relays. We employ the time-switching (TS) protocol, enabling the relays to harvest energy from the received previous hop signal to support data forwarding. We first prove that the system throughput monotonically increases with the transmit power of the source node. Next, by employing logarithmic transformations, we convert the non-convex problem of obtaining optimal TS ratios at each relay to maximize the system throughput into a convex optimization problem. Comprehensively taking into account the convergence rate, computational complexity per iteration, and robustness, we selected the log barrier method—a type of interior point method—to address this convex optimization problem, along with providing a detailed implementation procedure. The simulation results validate the optimality of the proposed method and demonstrate its applicability to practical communication systems. For instance, the proposed scheme achieves 1437.3 bps throughput at 40 dBm maximum source power in a 2-relay network—278.6% higher than that of the scheme with TS ratio fixed at 0.75 (379.68 bps). On the other hand, it converges within a 1.36 ms computation time for 5 relays, 6 orders of magnitude faster than exhaustive search (1730 s).

## 1. Introduction

### 1.1. Background

A wireless multi-hop relay network is a communication system that enables data forwarding from a source node to a destination node through the coordinated operation of multiple relay nodes. Its core technical concept involves replacing traditional single-hop links with flexible multi-segment short-hop links. This approach expands wireless communication coverage, addresses challenges caused by signal attenuation and interference, and ultimately enhances system capacity and energy efficiency. With remarkable flexibility in network configuration, this system is widely applicable to wireless sensor networks (WSNs), the Internet of Things (IoT), and emergency communication networks, and has emerged as one of the foundational solutions for these network paradigms [1,2]. For instance, when obstacles between the source and destination nodes prevent line-of-sight (LoS) transmission, relays can establish independent LoS links between consecutive hops in a multi-hop relay network. Although this approach requires additional communication time slots, it significantly enhances the signal-to-noise ratio (SNR) at the destination node.

In wireless multi-hop relay networks, powering relay nodes through built-in batteries introduces the challenge of significant labor costs associated with frequent battery replacements—an issue particularly pronounced in remote areas, industrial monitoring systems, or large-scale IoT deployments. Furthermore, battery lifespan is influenced by environmental temperature, depth of discharge, and other factors, adding complexity to network maintenance. Wireless energy harvesting (EH) technology stands as a robust candidate solution to address these challenges. By harnessing ambient radio frequency (RF) signals—a renewable energy source surrounding relay nodes—this technology enables relay nodes to achieve self-sustaining operation, thereby eliminating reliance on manual intervention [3,4]. Traditional EH technology focuses solely on extracting energy from the environment. Simultaneous wireless information and power transfer (SWIPT) is a subset of EH technology in the RF domain. The core innovation of SWIPT lies in its ability to leverage the same RF signal for simultaneous transmission of both information and energy [5,6]. By implementing SWIPT technology in multi-hop relay networks, relay nodes can assist the source node in delivering information to the destination node through hop-by-hop transmission without relying on batteries or external power supplies. That is, the relay nodes rely entirely on RF signal-based EH, with the harvested energy stored in integrated supercapacitors and fully utilized to power their information transmission to the next node during the information decoding (ID) phase.

Within the framework of SWIPT, the time-switching (TS) protocol and power-splitting (PS) protocol are two dominant approaches. Taking the TS protocol as an example, under this protocol, the receiver periodically toggles between EH and ID states via switching circuitry, thereby dividing each transmission slot into two subintervals: one dedicated to EH and the other to ID [7]. Their time allocation ratio, i.e., the TS ratio, is a critical parameter governing system performance trade-offs: extending the EH interval increases total harvested energy but simultaneously reduces the information transmission duration, leading to communication rate degradation. Therefore, balancing EH efficiency and communication quality by optimizing the TS ratio under the TS protocol or, correspondingly, optimizing the PS ratio under the PS protocol constitutes the core challenge in designing resource allocation algorithms for SWIPT systems [8]. Particularly in multi-hop relay networks with more than one relay node, the joint optimization of wireless resource allocation—including transmit power and multiple TS ratios or PS ratios—presents an even more intractable problem, which constitutes the primary research focus of this paper.

### 1.2. Related Work

In existing studies on SWIPT-enabled multi-hop relay networks, research efforts have primarily focused on optimizing specific performance metrics through resource allocation or analyzing system performance characteristics. The majority of early-stage investigations were concentrated on dual-hop relay networks employing amplify-and-forward (AF) or decode-and-forward (DF) relaying protocols [9,10,11,12,13,14,15,16]. For instance, reference [9] investigated the application of PS-SWIPT to establish a full-duplex dual-hop relay network where concurrent information reception and transmission are continuously maintained at the relay node. Compared with benchmark schemes, their proposed method more thoroughly utilizes the full-duplex characteristics, thereby significantly improving capacity. In [10], the authors investigated the secure relay beamforming problem for SWIPT in an AF two-way relay network. When the eavesdropper’s channel state information (CSI) is available, the optimization objective is to maximize the achievable secrecy sum rate under transmit power constraints and EH constraints. To reduce computational complexity, they proposed an iterative algorithm based on sequential parameter convex approximation to seek local optimal solutions. When the eavesdropper’s CSI is unavailable, they introduced an artificial noise-assisted secure relay beamforming scheme. In [11], the authors investigated the outage performance of PS-SWIPT relaying systems with a direct link between the source and destination. They first provided an outage probability analysis in closed-form expressions based on a high SNR approximation. It was demonstrated that the diversity order of SWIPT relaying systems remains identical to that of conventional non-SWIPT systems. The closed-form expressions further derived the closed-form solution for the optimal PS factor that minimizes the outage probability. In [12], the authors proposed a dynamic asymmetric PS scheme to minimize system outage probability by leveraging asymmetric instantaneous channel gains between the relay and destination nodes. They reformulated the intractable non-convex optimization problem into a fractional programming problem and developed a Dinkelbach-based iterative algorithm to find the optimal asymmetric PS ratios. Compared with existing static symmetric PS schemes, their proposed scheme with identical CSI overhead achieves a significant reduction in system outage probability. Reference [13] investigated the relay selection problem in full-duplex two-way relay networks, where relays are wirelessly powered by harvesting signal energy from source nodes. The authors demonstrated the quasi-convexity of the PS factor optimization problem and derived the optimal PS factor through linear search to minimize outage probability. Furthermore, corresponding relay selection schemes were developed for both single-relay scenarios and general cooperative scenarios with unrestricted relay quantities. In [14], the authors analyzed SWIPT-enabled battery-aided full-duplex relay networks, where relays supplement harvested energy with stored power to enhance reliability. By considering both TS and PS protocols, comparative analyses of outage probability and throughput were conducted for AF/DF relaying. For both AF/DF configurations, they identified a unique optimal battery energy threshold that maximizes throughput, establishing closed-form expressions for TS/PS parameters. An optimization framework was developed to prolong battery lifetime while achieving the target throughput.

In recent years, research on SWIPT-enabled multi-hop relay networks has shifted beyond single-relay scenarios to gradually encompass multi-relay configurations. However, studies specifically targeting multi-relay scenarios [17,18,19,20,21,22,23,24,25] remain significantly less prevalent compared to those focused on single-relay systems. As outlined in Section 1.1, resource allocation problems in multi-relay scenarios exhibit heightened complexity. This paper conducts a systematic investigation of current research advancements in this domain as follows. For instance, Reference [17] presented a pioneering investigation into multi-relay configurations, focusing on the DF protocol implementation in multi-hop wireless relay networks. This study established a SWIPT-enabled modeling framework incorporating a hybrid TS/PS protocol under fixed source transmit power with the objective of end-to-end throughput maximization. However, while having formulated protocol-specific optimization problems as convex programming models, this work provides no substantive derivation of solution methodologies. In [18], the authors presented a PS-SWIPT-enabled multi-hop DF relaying system with energy-autonomous relays. Employing convex optimization, the authors derived closed-form solutions for two core objectives: (1) minimum source power under node-specific quality of service (QoS) constraints, and (2) maximum achievable rate. Inspired by [18], the authors of [19] investigated the throughput optimization of industrial wireless sensor networks (IWSNs) with EH from interfering RF signals, considering the reliability constraints of industrial information transmission, and adopted the successive convex approximation (SCA) approach. Similarly, inspired by [18], the authors of [20] investigated a multi-hop multi-relay network in which all nodes are battery-assisted EH nodes that harvest energy from a power beacon. In each hop, the node that collects the most energy is selected for relaying and supplements the harvested energy with a portion of its battery energy. Considering the characteristics of nonlinear EH, the authors derived analytical expressions for outage probability, throughput, average battery energy consumption, and battery energy efficiency. In [21], the authors proposed a backpressure-based optimal joint routing and congestion control scheme for energy-autonomous wireless multi-hop networks. In [22], the authors developed a hybrid SWIPT protocol by merging TS and PS architectures, and designed a low-complexity routing scheme to optimize path energy efficiency. In Reference [23], the authors investigated the Age of Information (AoI) scheduling problem in multi-hop EH-WSNs. They designed the generation time of updates for nodes and developed transmission schedules under both the protocol interference model and the physical interference model, aiming to achieve the minimum peak AoI and average AoI for all nodes within a given duration. They proved that this problem is NP-hard and proposed an energy-adaptive, distributed algorithm suitable for general networks. Considering the relay’s energy-carrying capacity and security, the authors of [24] investigated the relay selection problem in energy-harvesting multi-hop device-to-device (D2D) networks with eavesdroppers. To enhance the secure connectivity performance of the relay, they derived the upper bound of the source-to-destination secrecy connectivity probability (SCP). Furthermore, they derived the factor directly related to the maximum SCP. Based on this, a relay selection algorithm was proposed to identify a relay path with higher security connectivity.

Moreover, emerging research paradigms have begun integrating deep learning architectures with multi-hop wireless relay systems, leveraging artificial intelligence (AI) advancements to address the complex resource allocation challenges inherent in these networks [25,26,27]. For example, Reference [25] presented a joint analytical-machine learning framework for multi-hop wirelessly powered IoT networks employing short-packet communications. Introducing a novel best relay-user selection protocol with accumulative energy harvesting, the authors established closed-form reliability-throughput tradeoffs under Rayleigh fading and developed deep neural network (DNN)-based real-time throughput predictors. In [27], the authors designed an efficient deep convolutional neural network (CNN) to improve and predict the performance of EH short-packet communications in multi-hop cognitive IoT networks. However, such AI-driven methodologies exhibit two critical limitations: (1) They rely on complex infrastructure and expensive hardware, typically requiring high-performance graphics processing units (GPUs) for the training and inference of DNNs, which escalates deployment costs and energy consumption beyond the practical constraints of resource-limited multi-hop networks. (2) They demand extensive datasets for training, necessitating the acquisition of historical CSI, traffic patterns, and EH profiles across diverse scenarios—these data acquisition challenges are particularly pronounced in dynamic wireless environments with non-stationary fading, often leading to potential overfitting or generalization failures in real-world deployments. Consequently, the practical application of AI-based approaches remains constrained by hardware overhead and data dependency, rendering them unsuitable for the low-cost, self-sustaining multi-hop relay systems emphasized in this work.

### 1.3. Motivation, Contribution, and Organization

According to the related work outlined in Section 1.2, current studies on SWIPT-enabled multi-hop relay networks primarily focus on dual-hop scenarios. Consequently, this study focuses on a SWIPT-enabled multi-hop relay network with multiple relays. Employing the DF protocol, the network implements a uniform single-antenna configuration across all nodal elements. In contrast to [17,18], the proposed system model employs a TS protocol, selected for its implementation advantages, including a simpler hardware structure, no requirement for power splitters, and lower implementation costs. Similar to [17,18], but unlike other studies mentioned above, the relay nodes in this model only collect energy from the RF signals transmitted by the previous node and store it in supercapacitors. All energy stored in the supercapacitors is used to forward decoded information to the next node. The typical application scenario of this model is when there is no LOS link between the source and destination nodes, and transmission is established through establishing LOS links over multiple hops. Furthermore, each node collects energy from the RF signals received from the previous node to power its own information signal transmission to the next node, requiring only the source node to be externally powered.

Research involving multiple relays emphasizes performance analysis [20,25,26] and resource allocation to optimize specific performance metrics [17,18,19,21,22,23,24,27]. The present study focuses on addressing the latter aspect by determining optimal solutions for both the source node’s transmission power and the TS ratios at each relay, aiming to maximize the system throughput. We first prove that the system throughput monotonically increases with the source node’s transmission power and that this throughput maximization problem constitutes a non-convex optimization problem. Subsequently, through logarithmic transformation techniques, we successfully convert this non-convex optimization problem into a convex formulation. Therefore, we are able to bypass computationally intensive SCA methods and AI-based optimization approaches that rely on expensive hardware and extensive training data. Different from Reference [17], which merely provided the proof that the system throughput maximization problem under a hybrid TS/PS protocol can be transformed into a convex optimization problem, we further propose a log barrier method-based approach featuring fast convergence, low computational complexity, and strong robustness—specifically, an interior-point iterative optimization algorithm for optimal solution derivation. The proposed log barrier method-based scheme demonstrates significant advantages over the Lagrangian dual method employed in [18] in terms of convergence rate and per-iteration computational complexity. For detailed comparative analysis, please refer to Section 4 of this paper.

WSNs often operate in energy-constrained environments (e.g., industrial IoT, environmental monitoring), where battery replacement is impractical. Our work on TS-SWIPT-enabled relays directly addresses this by enabling self-sustaining sensor nodes that harvest energy from RF signals while forwarding data. This is particularly critical for applications like disaster response networks or agricultural sensing, where multi-hop communication extends coverage without manual intervention.

The main contributions of this paper are summarized as follows:

1. We established a TS-SWIPT-based multi-hop DF relay network requiring only source node power supply, and converted the corresponding non-convex throughput maximization problem into a convex optimization formulation through logarithmic transformation.
2. By employing the log barrier method, the optimal solution of this convex optimization problem can be iteratively obtained with low computational complexity and a superlinear convergence rate.
3. Through comprehensive simulations, we validate the optimality of our proposed scheme. The system performance under various parameters is analyzed, with particular focus on the relationship between the number of relays and the system throughput. Furthermore, the algorithm’s rapid convergence characteristics confirm its practical viability for real-world communication systems.

The remainder of this paper is organized as follows: In Section 2, we present the system model of a multi-hop DF relay network enabled with TS-SWIPT and formulate a joint optimization problem aiming to maximize the system throughput by optimizing the transmit power at the source node and the TS ratio allocation at the relay nodes. Section 3 first proves that this problem is non-convex and then transforms it into a convex optimization problem via logarithmic transformations of the decision variables, along with rigorous convexity proofs. In Section 4, we compare three methods—interior-point method, exterior-point method, and Lagrangian dual method—for solving this convex problem. Section 5 provides a detailed iterative procedure for solving it using the log barrier method, a specialized interior-point method. Section 6 validates the optimality of our scheme through simulations, evaluates its performance, and demonstrates its applicability to practical communication systems. Finally, Section 7 concludes the paper.

## 2. System Model and Problem Formulation

**Figure 1:**
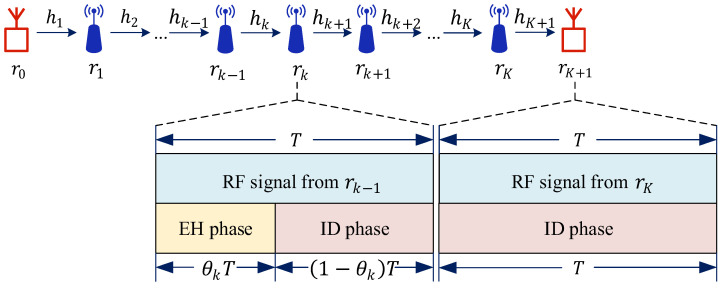
The multi-hop DF relay network with TS-SWIPT architecture.

Table 1 lists the main symbols first introduced in this section. The system model described in this section, as illustrated in Figure 1, comprises a multi-hop wireless relay network. The source node $r_{0}$ transmits data via K DF relays $\left\{r_{1},\ldots ,r_{K}\right\}$ over K+1 hops to the destination node $r_{K+1}$. We assume that SWIPT is applied exclusively between adjacent nodes during RF signal transmission. The channel coefficient between two adjacent nodes $r_{k-1}$ and $r_{k}$ at the k-th hop is denoted as $h_{k}$, $k\in \left\{1,\ldots ,K+1\right\}$, which remains constant during the EH and ID period T. Moreover, we assume non-adjacent channels exhibit sufficiently severe degradation—due to obstructions or deep fading—to be safely neglected [28]. This assumption draws from well-established routing scenarios in sensor networks where path selection algorithms are employed [29]. All the relay nodes are equipped with a single antenna and operate in half-duplex mode, meaning they relay forward received packets only after complete reception.

The proposed system model features the source node $r_{0}$ as the sole fixed-energy-supplied entity, while the relay node $r_{k}$, $k\in \left\{1,\ldots ,K\right\}$, relies entirely on RF signal-based EH. The EH and ID period T at $r_{k}$ is partitioned into two sequential phases: (1) EH phase, lasting $\theta_{k}T$, where $\theta_{k}\in \left(0,1\right)$ is the TS ratio, and (2) ID phase, lasting $(1-\theta_{k})T$. Particularly, at the destination node $r_{K+1}$, there is no EH phase, and the ID phase lasts for a duration of T.

Let $s_{k}$ denotes the information symbol at $r_{k}$, $k\in \left\{0,\ldots ,K\right\}$, where each symbol satisfies unit average power, i.e., $E\left[{\left|s_{k}\right|}^{2}\right]=1$. The received signal at $r_{k+1}$ can be expressed as:

(1) $$y_{k+1}=\sqrt{p_{k}}h_{k+1}s_{k}+n_{k+1},$$

where $p_{k}$ denotes the transmit power at $r_{k}$; $n_{k+1}$ represents the additive white Gaussian noise (AWGN) at $r_{k+1}$, and its power spectral density is assumed to be independent and identically distributed (i.i.d.) complex Gaussian random variables with zero mean and variance $\delta^{2}$, i.e., $n_{k+1}\sim CN\left(0,\delta^{2}\right)$.

For the EH phase depicted in Figure 1, the relay node $r_{k}$, $k\in \left\{1,\ldots ,K\right\}$ harvests energy from the RF signal $y_{k}$ over a duration of $\theta_{k}T$. The rectification efficiency of $r_{k}$ (the ratio of the energy harvester’s output power to input power) is denoted by $\eta_{k}$, where $\eta_{k}\in \left(0,1\right]$. Consequently, the harvested energy at relay node $r_{k}$, $k\in \left\{1,\ldots ,K\right\}$, is given by

(2) $$e_{k}=p_{k-1}g_{k}\eta_{k}\theta_{k}T,$$

where $g_{k}={\left|h_{k}\right|}^{2}$ denotes the channel gain between $r_{k-1}$ and $r_{k}$.

Assuming the relay node $r_{k}$ fully utilizes all the harvested energy $e_{k}$ during its ID period, the transmit power of $r_{k}$ is given by

(3) $$p_{k}=\frac{e_{k}}{T}=p_{k-1}g_{k}\eta_{k}\theta_{k}.$$

Since we can derive $p_{k-1}=p_{k-2}g_{k-1}\eta_{k-1}\theta_{k-1}$ from (3), by recursion, it follows that

(4) $$p_{k}=p_{0}\prod_{i=1}^{k}{g_{i}\eta }_{i}\theta_{i},$$

where $p_{0}$ denotes the transmit power of the source node $r_{0}$.

For ID, the received signal $y_{1}$ contains signal power $p_{0}g_{1}$ and noise power ${W\delta }^{2}$, where W denotes the transmission bandwidth. Thus, the received SNR at $r_{1}$ is expressed as

(5) $$\gamma_{1}=\frac{p_{0}g_{1}}{{W\delta }^{2}}=A_{1}p_{0},$$

where $A_{1}=\frac{g_{1}}{W\delta^{2}}$.

The received signal $y_{k}$, $k\in \left\{2,\ldots ,K+1\right\}$, contains signal power $p_{k-1}g_{k}$ and noise power ${W\delta }^{2}$. Thus, the received SNR at $r_{k}$ is expressed as

(6) $$\gamma_{k}=\frac{p_{k-1}g_{k}}{{W\delta }^{2}}=\frac{p_{0}\prod_{i=1}^{k}g_{i}\prod_{i=1}^{k-1}\eta_{i}\theta_{i}}{{W\delta }^{2}}=A_{k}p_{0}\prod_{i=1}^{k-1}\theta_{i},$$

where $A_{k}=\frac{\prod_{i=1}^{k}g_{i}\prod_{i=1}^{k-1}\eta_{i}}{{W\delta }^{2}}$.

Thus, the achievable data rate of hop k, $k\in \left\{1,\ldots ,K\right\}$ can be expressed as

(7) $$R_{k}=W\left(1-\theta_{k}\right)\ln\left(1+\gamma_{k}\right).$$

Specifically, as the destination node does not have an EH phase, as shown in Figure 1, the achievable data rate of hop K+1 can be represented as

(8) $$R_{K+1}=W\ln\left(1+\gamma_{K+1}\right).$$

Since the data transmission from the source node to the destination node undergoes K+1 EH and ID periods, the end-to-end achievable data rate in the multi-hop DF relay network, i.e., the system throughput, can be expressed as

(9) $$R=\frac{\min\left\{R_{1},\ldots R_{K+1}\right\}}{K+1}.$$

Within the TS protocol framework, the transmit power $p_{0}$ at the source node $r_{0}$ and the TS ratios $\theta_{k}$, $k\in \left\{1,\ldots ,K\right\}$ at each relay are configurable parameters.

This paper optimizes $p_{0}$ and $\theta_{k}$ to maximize the end-to-end achievable data rate R. Denoting $\theta =\left\{\theta_{k}|\theta_{k}\in \left(0,1\right),k=1,\ldots ,K\right\}$, the throughput maximization problem in our system model is formulated as

(10a) $$\underset{\theta ,p_{0}}{\max}R$$

(10b) $$s.t.\;0<\theta_{k}<1,\;k\in \left\{1,\ldots ,K\right\},$$

(10c) $$p_{min}\leq p_{0}\leq p_{max},$$

where $p_{min}$ and $p_{max}$ denote the minimum transmit power and maximum transmit power of the source node $r_{0}$, respectively.

## 3. Convex Optimization Problem Transformation

We list the main symbols first introduced in this section in Table 2. Benefiting from theoretical guarantees of global optimality and well-established efficient algorithms, convex optimization problems are generally more tractable in both theory and practice [30]. In this section, our primary focus is to reformulate Problem (10) as a convex optimization problem. First, we prove the following proposition.

**Proposition** **1.** *R is a monotonically increasing function with respect to $p_{0}$.*

**Proof** **of Proposition 1.** See Appendix A. □

The optimal solution for $p_{0}$ that maximizes R is $p_{max}$. Problem (10) can be reformulated as:

(11a) $$\underset{\theta }{\max}\min\left\{R_{1},\ldots R_{K+1}\right\}$$

(11b) $$s.t.\;0<\theta_{k}<1,\;k\in \left\{1,\ldots ,K\right\}.$$

Combining (5) to (8), the expressions for $R_{k}$, $k\in \left\{1,\ldots ,K+1\right\}$ in (11) can be rewritten as follows:

(12) $$R_{1}=W\left(1-\theta_{1}\right)\ln\left(1+B_{1}\right),$$

(13) $$R_{k}=W\left(1-\theta_{k}\right)\ln\left(1+B_{k}\prod_{i=1}^{k-1}\theta_{i}\right),\;k\in \left\{2,\ldots ,K\right\},$$

(14) $$R_{K+1}=W\ln\left(1+B_{K+1}\prod_{i=1}^{K}\theta_{i}\right),$$

where ${B_{k}=A}_{k}p_{max}$, $k\in \left\{1,\ldots ,K+1\right\}$.

According to the definition and judgment criteria of convex optimization problems [30], we can prove the following assumption:

**Proposition** **2.** *Problem (11) is not a convex optimization problem.*

**Proof** **of Proposition 2.** See Appendix B. □

To address the non-smoothness (non-differentiability) of the objective function $\min\left\{R_{1},\ldots R_{K+1}\right\}$ in Problem (11) and transform the complex min–max problem into a standard constrained optimization, we introduce an auxiliary variable $r=\frac{\min\left\{R_{1},\ldots R_{K+1}\right\}}{W}$. Problem (11) is thereby reformulated in the standard epigraph form [30]:

(15a) $$\underset{\theta ,r}{\max}r$$

(15b) $$s.t.\;\left(1-\theta_{1}\right)\ln\left(1+B_{1}\right)\geq r,$$

(15c) $$\left(1-\theta_{k}\right)\ln\left(1+B_{k}\prod_{i=1}^{k-1}\theta_{i}\right)\geq r,\;k\in \left\{2,\ldots ,K\right\},$$

(15d) $$\ln\left(1+B_{K+1}\prod_{i=1}^{K}\theta_{i}\right)\geq r,$$

(15e) $$0<\theta_{k}<1,\;k\in \left\{1,\ldots ,K\right\},$$

where (15b) denotes the first-hop constraint derived from (12), (15c) represents the intermediate-hop constraints derived from (13), and (15d) indicates the last-hop constraint derived from (14).

To transform the product term $\prod_{i=1}^{k-1}\theta_{i}$, $k\in \left\{2,\ldots ,K+1\right\}$ (appearing in (15c) and (15d)) into a tractable form, we introduce new variables $x_{k}=\ln\theta_{k}$ for $k\in \left\{1,\ldots ,K\right\}$. Since $0<\theta_{k}<1$, we have $x_{k}<0$. Given that the objective r>0 and the natural logarithm is monotonically increasing, maximizing r is equivalent to maximizing lnr. Define y=lnr and $x=\left\{x_{k}|x_{k}=\ln\theta_{k}\in \left(-\infty ,0\right),k=1,\ldots ,K\right\}$. Then, Problem (15) is reformulated into the standard form of convex optimization as follows:

(16a) $$\underset{x,y}{\min}-y$$

(16b) $$s.t.\;y-\ln\left(1-e^{x_{1}}\right)-\ln\left(\ln\left(1+B_{1}\right)\right)\leq 0,$$

(16c) $$y-\ln\left(1-e^{x_{k}}\right)-\ln\left(\ln\left(1+B_{k}e^{\sum_{i=1}^{k-1}x_{i}}\right)\right)\leq 0,\;k\in \left\{2,\ldots ,K\right\},$$

(16d) $$y-\ln\left(\ln\left(1+B_{K+1}e^{\sum_{i=1}^{K}x_{i}}\right)\right)\leq 0,$$

(16e) $$x_{k}<0,\;k\in \left\{1,\ldots ,K\right\}.$$

where (16b) denotes the transformed first-hop constraint derived from (15b), (16c) represents the transformed intermediate-hop constraints derived from (15c), and (16d) indicates the transformed last-hop constraint derived from (15d).

By the aforementioned transformation, Problem (10) is reformulated as a convex optimization problem. The proof is as follows.

**Proposition** **3.** *Problem (16) is a convex optimization problem.*

**Proof** **of Proposition 3.** See Appendix C. □

The convex transformation, as defined in (16), ensures computational efficiency, which is crucial for resource-constrained sensor nodes. For example, in structural health monitoring systems, sensors embedded in bridges or machinery require low-complexity algorithms to optimize energy usage while maintaining throughput.

## 4. Optimization Method Selection for Solving the Target Convex Problem

Since Problem (16) is a convex optimization problem, its local optimal solution is also the global optimal solution. This global optimum can be obtained via various common iterative methods, such as the interior point method, exterior point method, and Lagrangian dual method. We compare several mainstream methods for solving Problem (16) in terms of initial point requirements, convergence rate, and computational complexity [31]. We then summarize the results in Table 3.

Among these methods, the interior-point method converges rapidly, achieves superlinear convergence, and demonstrates strong robustness. Although inappropriate initial point selection may cause convergence failure, a feasible initial point can be obtained by choosing a feasible θ, which naturally aligns with the structure of Problem (16). Other methods for solving Problem (16) exhibit certain limitations, for instance:

Exterior point method: While flexible in initial point choice, its convergence rate is the slowest. Moreover, its penalty factor adjustment strategy easily triggers numerical ill-conditioning—excessively large factors cause deterioration of the Hessian matrix’s condition number, while overly small factors lead to severe constraint violations.

Lagrangian dual method: This method is only applicable to low-dimensional dual problems, i.e., when N is small and requires solving the dual problem via the subgradient method, resulting in a heavy computational burden and slow convergence with a sublinear convergence rate.

Therefore, we adopt a type of interior point method—the log barrier method—to obtain the global optimal solution for Problem (16). By transforming inequality constraints into penalty terms of the objective function, this method converts the original problem into a series of unconstrained optimization subproblems.

## 5. Optimal TS Ratio Allocation: A Log Barrier Approach

We list the main symbols first introduced in Section 4 and Section 5 in Table 4. This section delineates the detailed process of applying the interior-point barrier method to optimize TS ratio allocation as follows:

**Proposed algorithm.** Log barrier method for solving Problem (16).

### 5.1. Construction of the Barrier Problem

First, by utilizing functions (A6)–(A8) and $g_{k}\left(x_{k}\right)=x_{k}$, $k\in \left\{1,\ldots ,K\right\}$, we incorporate constraints (16b)–(16e) into the objective function in the form of log barrier terms. The constructed joint log barrier function is as follows:

(17) $$\phi \left(x,y\right)=-\sum_{k=1}^{K+1}\ln\left(-f_{k}\right)-\sum_{k=1}^{K}\ln\left(-g_{k}\right).$$

By introducing a barrier parameter t>0, the barrier objective function is defined as:

(18) $$\psi \left(x,y;t\right)=-y+\frac{1}{t}\phi \left(x,y\right).$$

The barrier problem is reformulated as:

(19) $$\left(x^{*},\;y^{*}\right)=\arg\underset{x,y}{\min}\psi \left(x,y;t\right).$$

Here, $\frac{1}{t}$ controls the weight of the barrier penalty term. As $\frac{1}{t}$ approaches zero from the positive side (t→+∞), the solution to the barrier problem (19) converges to the optimal solution of the original problem (16). In other words, since Problem (16) is convex and satisfies Slater’s condition, the log barrier method generates a continuous central path by solving a sequence of barrier subproblems (19) with increasing t, and converges to the global optimal solution along this path [31].

### 5.2. Initialization

The log barrier method consists of an outer loop and an inner loop. The outer loop updates the barrier parameter t, while the inner loop employs Newton’s method to solve the unconstrained optimization problem (19) with a fixed t. The process must ensure that the iterations remain strictly feasible at all times, i.e., satisfy (16b)–(16e).

Prior to commencing log barrier method iterations, we perform the following initialization routine:

Select an initial point $\left(x^{\left(0\right)},\;y^{\left(0\right)}\right)$ that satisfies the strictly feasible conditions (16b)–(16e).

Set the following parameters: An initial barrier parameter $t^{\left(0\right)}>0$; an update factor μ>1 for the barrier parameter t in the outer loop; a tolerance $\epsilon_{o}>0$ for the stopping criterion in the outer loop; a tolerance $\epsilon_{i}>0$ for the stopping criterion of Newton’s method in the inner loop.

Calculate the initial dual gap for the stopping criterion used in the outer loop: The duality gap in the log barrier method is approximately $\frac{m}{t}$, where m denotes the total number of constraints in Problem (16). Since the total number of constraints is 2K+1, the initial duality gap is $\frac{2K+1}{t^{\left(0\right)}}$.

### 5.3. Outer Loop

Within each outer iteration of the log barrier method, the following sequence is executed:

#### 5.3.1. Check the Stopping Criteria in the Outer Loop

For the current $t^{\left(n\right)}$, where n is a non-negative integer, if the duality gap is less than the tolerance of the outer loop, i.e., $\frac{2K+1}{t^{\left(n\right)}}<\epsilon_{o}$, terminate the outer loop; otherwise, the algorithm proceeds to the inner loop of the log barrier method as follows.

#### 5.3.2. Inner Loop

Fix $t=t^{\left(n\right)}$ and solve Subproblem (19) with the current $\left(x^{\left(n\right)},\;y^{\left(n\right)}\right)$ as the initial value via Newton’s method. We repeat the following seven steps until inner loop convergence is achieved. First, the gradient vector and Hessian matrix of the barrier objective function $\psi \left(x,y;t\right)$ are computed.

1 Gradient computation

To obtain the gradient $\nabla \psi ={\left[\frac{\partial \psi }{\partial x_{1}},\ldots ,\frac{\partial \psi }{\partial x_{K}},\frac{\partial \psi }{\partial y}\right]}^{T}$, we proceed with the following calculations.

For the element $\frac{\partial \psi }{\partial x_{j}}$, $j\in \left\{1,\ldots ,K\right\}$, in the gradient ∇ψ, we have

(20) $$\frac{\partial \psi }{\partial x_{j}}=\frac{1}{t}\left(\sum_{k=1}^{K+1}\frac{1}{f_{k}}\frac{\partial f_{k}}{\partial x_{j}}+\frac{1}{x_{j}}\right),$$

where the calculation of $\frac{\partial f_{k}}{\partial x_{j}}$ is categorized into the following cases:

Case 1: When k=1,

(21) $$\frac{\partial f_{1}}{\partial x_{j}}=\left\{\begin{array}{l} \frac{e^{x_{1}}}{1-e^{x_{1}}},\;j=1\\ 0,\;j\neq 1 \end{array}\right..$$

Case 2: When $k\in \left\{2,\ldots ,K\right\}$,

(22) $$\frac{\partial f_{k}}{\partial x_{j}}=\left\{\begin{array}{l} -\frac{B_{k}e^{\sum_{i=1}^{k-1}x_{i}}}{\ln\left(1+B_{k}e^{\sum_{i=1}^{k-1}x_{i}}\right)\left(1+B_{k}e^{\sum_{i=1}^{k-1}x_{i}}\right)},\;j<k\\ \frac{e^{x_{k}}}{1-e^{x_{k}}},\;j=k\\ 0,\;j>k \end{array}\right..$$

Case 3: When k=K+1,

(23) $$\frac{\partial f_{K+1}}{\partial x_{j}}=-\frac{B_{K+1}e^{\sum_{i=1}^{K}x_{i}}}{\ln\left(1+B_{K+1}e^{\sum_{i=1}^{K}x_{i}}\right)\left(1+B_{K+1}e^{\sum_{i=1}^{K}x_{i}}\right)}.$$

For the element $\frac{\partial \psi }{\partial y}$ in the gradient ∇ψ, we have

(24) $$\frac{\partial \psi }{\partial y}=-1-\frac{1}{t}\sum_{k=1}^{K+1}\frac{1}{f_{k}}.$$

2 Hessian matrix computation

The Hessian matrix $\nabla^{2}\psi$ has a dimension of $\left(K+1\right)\times \left(K+1\right)$, and its complete matrix form is expressed as follows:

(25) $$\nabla^{2}\psi =\left[\begin{array}{ccccc} \frac{\partial^{2}\psi }{\partial x_{1}^{2}} & \frac{\partial^{2}\psi }{\partial x_{1}\partial x_{2}} & \ldots & \frac{\partial^{2}\psi }{\partial x_{1}\partial x_{K}} & \frac{\partial^{2}\psi }{\partial x_{1}\partial y}\\ \frac{\partial^{2}\psi }{\partial x_{2}\partial x_{1}} & \frac{\partial^{2}\psi }{\partial x_{2}^{2}} & \ldots & \frac{\partial^{2}\psi }{\partial x_{2}\partial x_{K}} & \frac{\partial^{2}\psi }{\partial x_{2}\partial y}\\ \vdots & \vdots & \ddots & \vdots & \vdots \\ \frac{\partial^{2}\psi }{\partial x_{K}\partial x_{1}} & \frac{\partial^{2}\psi }{\partial x_{K}\partial x_{2}} & \ldots & \frac{\partial^{2}\psi }{\partial x_{K}^{2}} & \frac{\partial^{2}\psi }{\partial x_{K}\partial y}\\ \frac{\partial^{2}\psi }{\partial y\partial x_{1}} & \frac{\partial^{2}\psi }{\partial y\partial x_{2}} & \ldots & \frac{\partial^{2}\psi }{\partial y\partial x_{K}} & \frac{\partial^{2}\psi }{\partial y^{2}} \end{array}\right].$$

where its elements are calculated as follows:

For the diagonal element $\frac{\partial^{2}\psi }{\partial x_{j}^{2}}$ of the Hessian matrix in (25), we have

(26) $$\frac{\partial^{2}\psi }{\partial x_{j}^{2}}=\frac{1}{t}\left\{\sum_{k=1}^{K+1}\left[\frac{1}{f_{k}^{2}}{\left(\frac{\partial f_{k}}{\partial x_{j}}\right)}^{2}-\frac{1}{f_{k}}\frac{\partial^{2}f_{k}}{\partial x_{j}^{2}}\right]-\frac{1}{x_{j}^{2}}\right\}.$$

For the term $\frac{\partial f_{k}}{\partial x_{j}}$ in (26), we can compute it using (21)–(23). As for the second-order derivative $\frac{\partial^{2}f_{k}}{\partial x_{j}^{2}}$ in (26), we perform case-specific calculations as follows:

Case 1: When k=1,

(27) $$\frac{\partial^{2}f_{1}}{\partial x_{j}^{2}}=\left\{\begin{array}{l} \frac{e^{x_{1}}}{{\left(1-e^{x_{1}}\right)}^{2}},\;j=1\\ 0,\;j\neq 1 \end{array}\right..$$

Case 2: When $k\in \left\{2,\ldots ,K\right\}$,

(28) $$\frac{\partial^{2}f_{k}}{\partial x_{j}^{2}}=\left\{\begin{array}{l} -\frac{B_{k}e^{\sum_{i=1}^{k-1}x_{i}}\left[\ln\left(1+B_{k}e^{\sum_{i=1}^{k-1}x_{i}}\right)\left(1+B_{k}e^{\sum_{i=1}^{k-1}x_{i}}\right)-B_{k}e^{\sum_{i=1}^{k-1}x_{i}}\right]}{{\left[\ln\left(1+B_{k}e^{\sum_{i=1}^{k-1}x_{i}}\right)\left(1+B_{k}e^{\sum_{i=1}^{k-1}x_{i}}\right)\right]}^{2}},\;j<k\\ \frac{e^{x_{k}}}{{\left(1-e^{x_{k}}\right)}^{2}},\;j=k\\ 0,\;j>k \end{array}\right..$$

Case 3: When k=K+1,

(29) $$\frac{\partial^{2}f_{K+1}}{\partial x_{j}^{2}}=-\frac{B_{K+1}e^{\sum_{i=1}^{K}x_{i}}\left[\ln\left(1+B_{K+1}e^{\sum_{i=1}^{K}x_{i}}\right)\left(1+B_{K+1}e^{\sum_{i=1}^{K}x_{i}}\right)-B_{K+1}e^{\sum_{i=1}^{K}x_{i}}\right]}{{\left[\ln\left(1+B_{K+1}e^{\sum_{i=1}^{K}x_{i}}\right)\left(1+B_{K+1}e^{\sum_{i=1}^{K}x_{i}}\right)\right]}^{2}}.$$

For the diagonal element $\frac{\partial^{2}\psi }{\partial y^{2}}$ of the Hessian matrix in (25), we have

(30) $$\frac{\partial^{2}\psi }{\partial y^{2}}=\frac{1}{t}\sum_{k=1}^{K+1}\frac{1}{f_{k}^{2}}.$$

For the off-diagonal element $\frac{\partial^{2}\psi }{\partial x_{j}\partial x_{l}}$ ($j\in \left\{1,\ldots ,K\right\}$, $l\in \left\{1,\ldots ,K\right\}$ and j≠l) of the Hessian matrix in (25), we have

(31) $$\frac{\partial^{2}\psi }{\partial x_{j}\partial x_{l}}=\frac{1}{t}\sum_{k=1}^{K+1}\left[\frac{1}{f_{k}^{2}}\frac{\partial f_{k}}{\partial x_{j}}\frac{\partial f_{k}}{\partial x_{l}}-\frac{1}{f_{k}}\frac{\partial^{2}f_{k}}{\partial x_{j}\partial x_{l}}\right].$$

For the term $\frac{\partial f_{k}}{\partial x_{j}}\frac{\partial f_{k}}{\partial x_{l}}$ in (31), we compute it by considering different cases as follows:

Case 1: When k=1,

(32) $$\frac{\partial f_{1}}{\partial x_{j}}\frac{\partial f_{1}}{\partial x_{l}}=0.$$

Case 2: When $k\in \left\{2,\ldots ,K\right\}$,

(33) $$\frac{\partial f_{k}}{\partial x_{j}}\frac{\partial f_{k}}{\partial x_{l}}=\left\{\begin{array}{l} {\left[\frac{B_{k}e^{\sum_{i=1}^{k-1}x_{i}}}{\ln\left(1+B_{k}e^{\sum_{i=1}^{k-1}x_{i}}\right)\left(1+B_{k}e^{\sum_{i=1}^{k-1}x_{i}}\right)}\right]}^{2},\;j<k\;\mathrm{and}\;l<k\\ -\frac{e^{x_{k}}}{1-e^{x_{k}}}\cdot \frac{B_{k}e^{\sum_{i=1}^{k-1}x_{i}}}{\ln\left(1+B_{k}e^{\sum_{i=1}^{k-1}x_{i}}\right)\left(1+B_{k}e^{\sum_{i=1}^{k-1}x_{i}}\right)},\;j=k\;\mathrm{and}\;l<k\\ -\frac{e^{x_{k}}}{1-e^{x_{k}}}\cdot \frac{B_{k}e^{\sum_{i=1}^{k-1}x_{i}}}{\ln\left(1+B_{k}e^{\sum_{i=1}^{k-1}x_{i}}\right)\left(1+B_{k}e^{\sum_{i=1}^{k-1}x_{i}}\right)},\;j<k\;\mathrm{and}\;l=k\\ 0,\;j>k\;\mathrm{or}\;l>k \end{array}\right..$$

Case 3: When k=K+1,

(34) $$\frac{\partial f_{K+1}}{\partial x_{j}}\frac{\partial f_{K+1}}{\partial x_{l}}={\left[\frac{B_{K+1}e^{\sum_{i=1}^{K}x_{i}}}{\ln\left(1+B_{K+1}e^{\sum_{i=1}^{K}x_{i}}\right)\left(1+B_{K+1}e^{\sum_{i=1}^{K}x_{i}}\right)}\right]}^{2}.$$

For the term $\frac{\partial^{2}f_{k}}{\partial x_{j}\partial x_{l}}$ in (31), we compute it by considering different cases as follows:

Case 1: When k=1,

(35) $$\frac{\partial^{2}f_{1}}{\partial x_{j}\partial x_{l}}=0.$$

Case 2: When $k\in \left\{2,\ldots ,K\right\}$,

(36) $$\frac{\partial^{2}f_{k}}{\partial x_{j}\partial x_{l}}=\left\{\begin{array}{l} -\frac{B_{k}e^{\sum_{i=1}^{k-1}x_{i}}\left[\ln\left(1+B_{k}e^{\sum_{i=1}^{k-1}x_{i}}\right)-B_{k}e^{\sum_{i=1}^{k-1}x_{i}}\right]}{{\left[\ln\left(1+B_{k}e^{\sum_{i=1}^{k-1}x_{i}}\right)\left(1+B_{k}e^{\sum_{i=1}^{k-1}x_{i}}\right)\right]}^{2}},\;j<k\;\mathrm{and}\;l<k\\ 0,\;j=k\;\mathrm{and}\;l<k\\ 0,\;j<k\;\mathrm{and}\;l=k\\ 0,\;j>k\;\mathrm{or}\;l>k \end{array}\right..$$

Case 3: When k=K+1,

(37) $$\frac{\partial^{2}f_{K+1}}{\partial x_{j}\partial x_{l}}=-\frac{B_{K+1}e^{\sum_{i=1}^{K}x_{i}}\left[\ln\left(1+B_{K+1}e^{\sum_{i=1}^{K}x_{i}}\right)-B_{K+1}e^{\sum_{i=1}^{K}x_{i}}\right]}{{\left[\ln\left(1+B_{K+1}e^{\sum_{i=1}^{K}x_{i}}\right)\left(1+B_{K+1}e^{\sum_{i=1}^{K}x_{i}}\right)\right]}^{2}}.$$

For the off-diagonal element $\frac{\partial^{2}\psi }{\partial x_{j}\partial y}$ of the Hessian matrix in (25), we have

(38) $$\frac{\partial^{2}\psi }{\partial x_{j}\partial y}=\frac{1}{t}\sum_{k=1}^{K+1}\frac{1}{f_{k}^{2}}\frac{\partial f_{k}}{\partial x_{j}},$$

where the term $\frac{\partial f_{k}}{\partial x_{j}}$ can be obtained from (21)–(23).

With ∇ψ and $\nabla^{2}\psi$ computed, we proceed to implement Newton’s method within the inner loop of our scheme based on the log barrier method. Subsequently, the Newton direction and Newton decrement expressions are presented as follows.

3 Newton direction computation

When solving the unconstrained optimization problem (19), Newton’s method requires computing a search direction, known as the Newton direction [31]. Based on the obtained gradient ∇ψ and the Hessian matrix $\nabla^{2}\psi$, we can derive the Newton direction ${\left[∆x,∆y\right]}^{T}$ by solving the following linear system:

(39) $$\nabla^{2}\psi \cdot {\left[∆x,∆y\right]}^{T}=-\nabla \psi ,$$

where $∆x=\left[{∆x}_{1},\ldots {,∆x}_{K}\right]$ is the step size for variable vector x, and ∆y denotes the step size of variable y.

4 Newton decrement computation

The Newton decrement is a key convergence metric in Newton’s method, used to measure the distance from the current point to the local optimal solution (since Problem (16) has been proven to be a convex optimization problem, the local optimum coincides with the global optimum). In the inner loop (Newton iterations) of the interior-point method, it serves as a stopping criterion to determine when to terminate the Newton iterations under the current barrier parameter t [31]. Using the obtained ${\left[∆x,∆y\right]}^{T}$ from above, the Newton decrement can be indirectly computed as follows:

(40) $$\lambda =\sqrt{-{\nabla \psi }^{T}{\left[∆x,∆y\right]}^{T}}=\sqrt{{\nabla \psi }^{T}\nabla^{2}\psi^{-1}\nabla \psi }.$$

5 Backtracking Line Search

In this step, we employ a backtracking line search to obtain a step size α that ensures strict feasibility and sufficient decrease for x and y during the update process. First, we set α=1. If any of the following two conditions is not satisfied, update α with αβ, namely, α←αβ, where β is a constant with $\beta \in \left(0,1\right)$, typically taken as 0.5:

**Strict feasibility condition:** In iterative optimization, the step size must be chosen such that all iterates lie strictly inside the feasible domain (satisfying constraints exactly), preventing encroachment on constraint boundaries. That is to say, α must satisfy

(41) $$f_{1}\left(x_{1}+\alpha {∆x}_{1},y+\alpha ∆y\right)<0,$$

(42) $$f_{k}\left(x_{1}+\alpha {∆x}_{1},\ldots ,x_{k}+\alpha {∆x}_{k},y+\alpha ∆y\right)<0,\;k\in \left\{2,\ldots ,K\right\},$$

(43) $$f_{K+1}\left(x_{1}+\alpha {∆x}_{1},\ldots ,x_{K}+\alpha {∆x}_{K},y+\alpha ∆y\right)<0.$$

(44) $$g_{k}\left(x_{k}+\alpha {∆x}_{k}\right)<0,\;k\in \left\{1,\ldots ,K\right\}.$$

**Armijo condition (sufficient decrease):** Ensure that the value of the objective function ψ is sufficiently decreased during iterations; i.e., parameter α needs to satisfy

(45) $$\psi \left(x+\alpha ∆x,y+\alpha ∆y;t\right)\leq \psi \left(x,y;t\right)+c\alpha \nabla \psi^{T}{\left[∆x,∆y\right]}^{T},$$

where c is a constant with $c\in \left(0,1\right)$, typically taken as $10^{-4}$.

6 Update the primal variables

Based on the α obtained in Step 5, we update the x and y in the inner loop, i.e., x←x+α∆x and y←y+α∆y.

7 Check whether the inner loop should stop

At this stage, we compute the Newton decrement λ by substituting the updated x and y into (40). Subsequently, if the condition $\frac{\lambda^{2}}{2}<\epsilon_{i}$ is satisfied, we terminate the inner loop and then obtain the updated primal variables $\left(x^{\left(n+1\right)},\;y^{\left(n+1\right)}\right)$.

#### 5.3.3. Update the Barrier Parameter

After performing the above operations, we have obtained the independent variables $\left(x^{\left(n+1\right)},\;y^{\left(n+1\right)}\right)$. Then, we can update the barrier parameter $t^{\left(n+1\right)}$ for outer loop n+1 as $t^{\left(n+1\right)}\leftarrow {\mu t}^{\left(n\right)}$.

The algorithm then returns to Section 5.3.1 and iterates until it outputs the optimal solution $\left(x^{*},\;y^{*}\right)$. Upon obtaining the optimal solution $x^{*}$, through the transformation $\theta_{k}=e^{x_{k}}$ for k=1,…,K, we derive the optimal TS ratios that maximize the throughput of our SWIPT-enabled multi-hop DF relay network. According to [31], our interior-point barrier method-based scheme achieves a convergence rate of $O\left(\sqrt{N}\log\frac{1}{\epsilon_{o}}\right)$ with per-iteration computational cost of $O\left(N^{3}\right)$, resulting in an overall complexity $O\left(N^{3.5}\log\frac{1}{\epsilon_{o}}\right)$. For clarity, the complete workflow of the proposed algorithm is presented in Algorithm 1.
**Algorithm 1.** The proposed algorithm**Begin** Construct joint log barrier function $\phi \left(x,y\right)$ Construct barrier objective function $\psi \left(x,y;t\right)$
 **Initialization:**  Set strictly feasible initial point $\left(x^{\left(0\right)},\;y^{\left(0\right)}\right)$ satisfying (16b)–(16e)  Set initial barrier parameter $t^{\left(0\right)}>0$, update factor μ>1, tolerance $\epsilon_{o}>0$, Newton tolerance $\epsilon_{i}>0$
 **while** $\frac{2K+1}{t^{\left(n\right)}}<\epsilon_{o}$ do  ▷ *Outer loop: Barrier parameter update*  Set Newton initial point $\left(x,y\right)=\left(x^{\left(n\right)},\;y^{\left(n\right)}\right)$  **repeat**  **▷** *Inner loop: Newton’s method*   Compute gradient ∇ψ
   Compute Hessian matrix $\nabla^{2}\psi$
   Solve Newton system $\nabla^{2}\psi \cdot {\left[∆x,∆y\right]}^{T}=-\nabla \psi$ for search direction ${\left[∆x,∆y\right]}^{T}$
   Compute Newton decrement $\lambda =\sqrt{-{\nabla \psi }^{T}{\left[∆x,∆y\right]}^{T}}$
   Set backtracking line search parameters α=1, β=0.5, $c=10^{-4}$
   **while** true do  ▷ *Backtracking line search*    **if** $\left(x+\alpha ∆x,y+\alpha ∆y\right)$ strictly feasible and $\psi \left(x+\alpha ∆x,y+\alpha ∆y;t\right)\leq \psi \left(x,y;t\right)+c\alpha \nabla \psi^{T}{\left[∆x,∆y\right]}^{T}$
     **break**    **else**     **Update** α←αβ
    **end if**   **end while**   **Update** x←x+α∆x and y←y+α∆y  **until** $\frac{\lambda^{2}}{2}<\epsilon_{i}$  ▷ *Newton convergence criteria*  **Update** $\left(x^{\left(n+1\right)},\;y^{\left(n+1\right)}\right)\leftarrow \left(x,y\right)$  ▷ *Prepare for next outer iteration*  **Update** $t^{\left(n+1\right)}\leftarrow {\mu t}^{\left(n\right)}$  ▷ *Increase barrier parameter* **end while** Output optimal solution $\left(x^{*},\;y^{*}\right)$
**End**

## 6. Simulation Results and Discussion

This section evaluates the performance of the proposed scheme in our SWIPT multi-hop DF relay network model through simulation. All simulations were executed on a laptop equipped with an AMD Ryzen R9 7945HX CPU and 64 GB RAM. The software environment utilized MATLAB R2022b for algorithm implementation and data analysis.

All simulations in this section use the following parameter settings unless otherwise specified: $\delta^{2}=-174$ dBm/Hz; the rectification efficiency of each relay node is set to 0.9, namely, $\eta_{k}=0.9$ for $k\in \left\{1,\ldots ,K\right\}$; W=1 MHz; $t^{\left(0\right)}=1$; μ=10; $\epsilon_{o}=10^{-6}$; $\epsilon_{i}=10^{-4}$; the initial value of α is set to 1; β=0.5; $c=10^{-4}$. Since it has been proven in Section 3 that the system throughput monotonically increases with the transmit power $p_{0}$ of the source node, it is assumed that the source node transmits at the maximum power $p_{max}$. Moreover, it is assumed that in our multi-hop relay network, the distance between each hop is equal, and the total distance of all hops sums to 10 m.

In terms of channel modeling, for large-scale fading, a log-distance path loss model with a path loss exponent of 3.8 is adopted, where the carrier frequency is 2.4 GHz, and the reference distance is set to 1m. For small-scale fading, LoS links are established between the transmitting and receiving nodes at each hop via relay deployment. Therefore, the channel coefficient $h_{k}$ for each hop follows a Rician fading distribution with a K-factor of 7. The simulation results are generated by averaging over 1000 random channel realizations.

The proposed scheme, based on the log barrier method, is compared against the following benchmark schemes: one is the performance-optimal exhaustive search-based scheme, employing a TS ratio search step size of 0.01; the others are those with identical TS ratios fixed at all relays, specifically at 0.25, 0.5, and 0.75.

### 6.1. Throughput vs. Maximum Source Transmit Power

**Figure 2:**
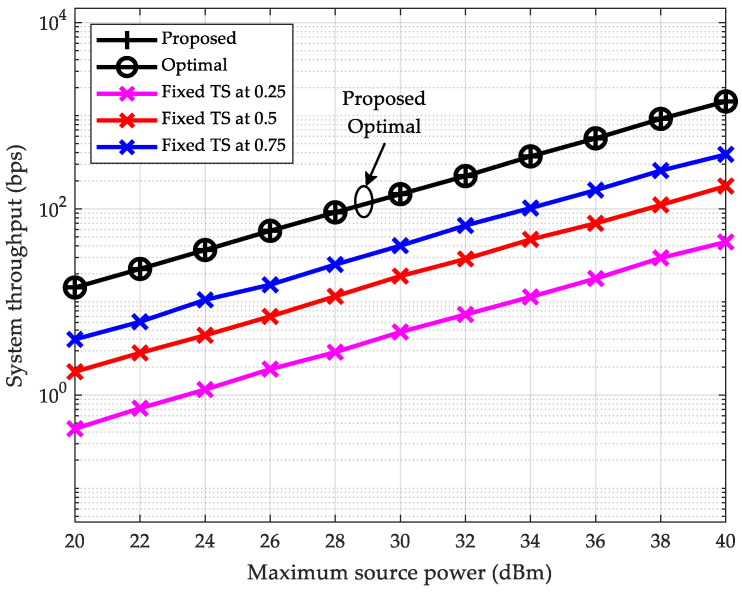
System throughput vs. maximum source power (K=2).

First, in Figure 2, we plot the relationship between the end-to-end throughput and the maximum transmit power of the source node $p_{max}$ for the SWIPT-enabled multi-hop DF relay network. The range of $p_{max}$ is from 20 dBm to 40 dBm. The hop count of the multi-hop relay network is set to K=2; thus, the per-hop distance is $\frac{10}{K+1}=3.33$ m. In this figure, it can be clearly observed that the throughput of all schemes increases with the maximum transmit power of the source node. For instance, the throughput of the proposed scheme increases from 14.29 bps at $p_{max}=20$ dBm to 1420.45 bps at $p_{max}=40$ dBm. Moreover, the curves of the proposed scheme based on the log barrier method and the exhaustive search-based scheme coincide, indicating identical throughput performance. When tested under other configurations, the curves remain coincident, demonstrating the optimality of the proposed scheme. Additionally, as expected, the proposed scheme significantly outperforms fixed TS ratio schemes in throughput performance. Additional simulations confirm that this advantage persists when TS ratios are fixed at other values. The scheme with TS ratios fixed at 0.87 at all relays exhibits the smallest performance gap from the proposed (optimal) scheme. Even then, its throughput only reaches approximately 47.3% of that of the proposed (optimal) scheme on average. This fully demonstrates the necessity of implementing the proposed scheme to fully exploit the performance potential in our SWIPT-enabled multi-hop DF relay network.

### 6.2. Throughput vs. Number of Relays

**Figure 3:**
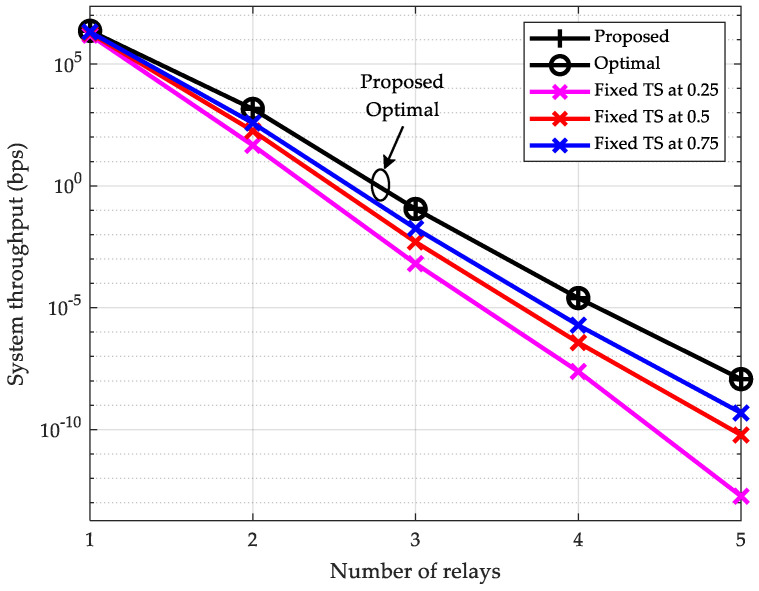
System throughput vs. number of relays ($p_{max}=40$ dBm).

Next, we examine the relationship between system throughput and the number of relays. The maximum transmit power of the source node, $p_{max}$, is fixed at 40 dBm. The number of relays, K, in the multi-hop relay network increases from 1 to 5, corresponding to a hop count growth from 2 to 6 between the source and destination. Given equal per-hop distance and a total distance of 10 m for all hops, the resulting per-hop distances are 5 m, 3.33 m, 2.5 m, 2 m, and 1.67 m, respectively. Figure 3 plots the system throughput versus the number of relays K. From Figure 3, conclusions analogous to those in Figure 2 can be drawn: the proposed scheme possesses optimal performance, and its throughput significantly outperforms that of the fixed TS ratio schemes. For instance, when the number of relays is set to K=2, the throughput of both the proposed scheme and the exhaustive search-based scheme reaches an identical value of 1437.3 bps, while that of the scheme with TS ratio fixed at 0.75 is only 379.68 bps. On the other hand, it can be observed that the throughput of all schemes declines rapidly with the increase in the number of relays. Taking the proposed scheme as an example, when K=1, its throughput is $2.31\times 10^{6}$ bps, but when K=5, its throughput drops to merely $1.17\times 10^{-8}$ bps, becoming negligible. This phenomenon can be attributed to Equation (9): as the number of relays increases, transmitting constant-size data requires more EH and ID periods. Furthermore, the system throughput is constrained by the hop with the minimum achievable data rate. This insight suggests that when channel conditions between the source and destination nodes are poor and the SWIPT-enabled multi-hop DF relay network is deployed, the number of hops should be minimized under the premise that favorable channel conditions can be achieved at each hop. Nevertheless, it is also observable that the throughput of the proposed scheme declines more slowly than the fixed TS ratio schemes as the number of relays increases, particularly when the number of relays is high. This further demonstrates the superiority of the proposed scheme.

### 6.3. Throughput vs. Rectification Efficiency

**Figure 4:**
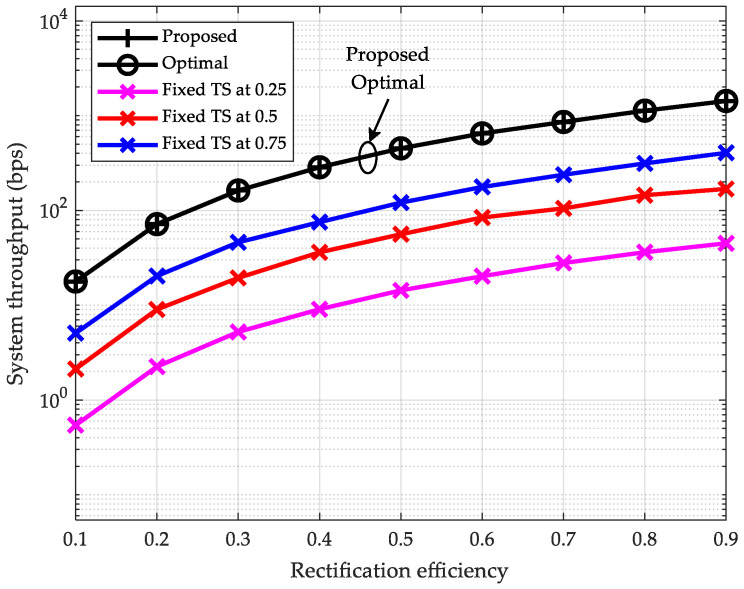
System throughput vs. rectification efficiency (K=2, $p_{max}=40$ dBm).

In Figure 4, we compare the variation trends of the system throughput under varying rectification efficiencies. The rectification efficiency is set to increase from 0.1 to 0.9, with the number of relays between source and destination nodes fixed at K=2 and $p_{max}=40$ dBm. Similar to the aforementioned scenarios, the proposed scheme delivers optimal performance and is significantly superior to the fixed TS ratio schemes. Illustrated by the case where $\eta_{k}=0.5$, the throughput of the proposed scheme is approximately 32 times that of the scheme with the TS ratio fixed at 0.25. Additionally, as the rectification efficiency $\eta_{k}$ increases from 0.1 to 0.9, the system throughput of the proposed scheme rises from 17.79 bps to 1426.72 bps, representing an improvement by approximately a factor of 80. It follows that a higher rectification efficiency enables the multi-hop relay network architecture to accommodate more relays.

### 6.4. Computational Efficiency Analysis

**Figure 5:**
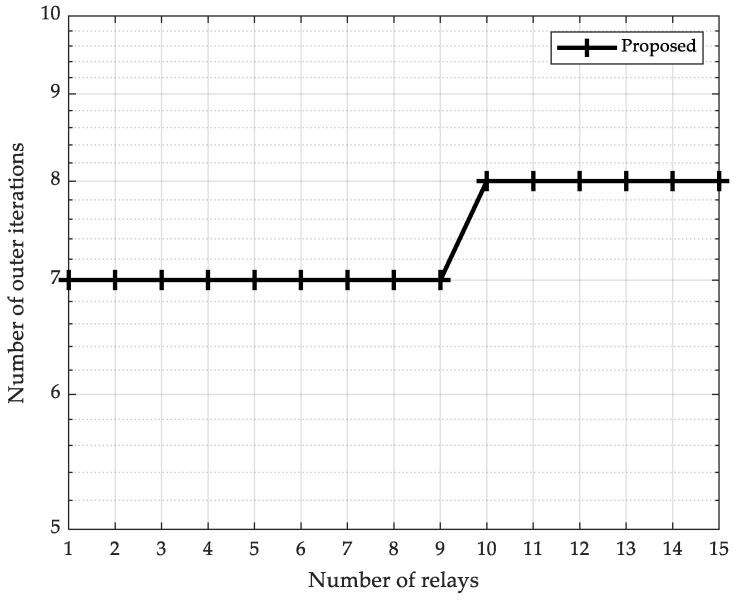
Number of outer iterations vs. number of relays ($p_{max}=40$ dBm).

The preceding simulation results have demonstrated the throughput performance of the proposed scheme. We now proceed to examine its execution efficiency. We set $p_{max}=40$ dBm and vary the number of relays K from 1 to 15. Figure 5 demonstrates the number of outer iterations versus the number of relays K for the proposed scheme based on the log barrier method. Notably, under typical relay deployments (K<10), the proposed scheme consistently converges within 7 outer iterations. This signifies that the proposed scheme achieves convergence with fewer iterations, and its iteration count remains nearly constant as the scale of the multi-hop relay network increases.

**Figure 6:**
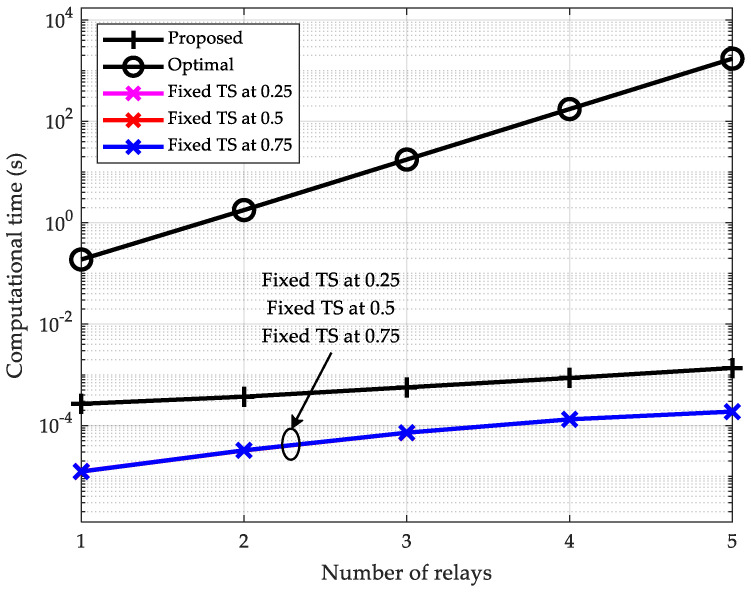
Computational time vs. number of relays ($p_{max}=40$ dBm).

In Figure 6, to further investigate the computational efficiency of the proposed scheme, we measure the computational time of all schemes. From this figure, it is observed that the computational time of the optimal exhaustive search-based scheme increases prohibitively with the number of relays. When K=5, this scheme requires 1730 s to complete execution, which is a prohibitively high latency for practical communication systems. Conversely, and as anticipated, the three fixed-TS schemes exhibit nearly identical and minimal computation times. For instance, when K=5, execution completes in just $1.88\times 10^{-4}$ s. Notably, the proposed scheme exhibits computation times only one order of magnitude higher than the fixed-TS schemes. Specifically, when K=5, it requires merely $1.36\times 10^{-3}$ s. Furthermore, mirroring the characteristics shown in Figure 5, the proposed scheme exhibits comparatively gradual computation time growth with increasing relay nodes.

The above results conclusively demonstrate that the proposed scheme not only delivers optimal performance but also achieves rapid convergence with modest computational demands, making it highly suitable for real-time deployment.

## 7. Conclusions

This paper establishes a novel convex optimization framework for maximizing end-to-end throughput in multi-hop DF relay networks employing the TS-SWIPT protocol. The originally nonconvex problem was transformed into a tractable convex form via logarithmic conversion, enabling the application of the log barrier method to derive the globally optimal TS ratios. Numerical simulations validate the proposed method’s dual merits: optimal performance and rapid convergence, demonstrating its applicability to practical communication systems. Specifically, the proposed scheme achieves a throughput of 1437.3 bps in a 3-hop relay network at 40 dBm maximum source transmit power, which is 278% higher than that of the fixed TS scheme at 0.75, and it exhibits millisecond-level convergence time. By enabling joint optimization of the TS ratios across relays, our system supports self-sustaining, battery-free relay operation, eliminating battery replacement costs for deployment in remote areas, industrial IoT, and emergency communications. This work provides a computationally efficient framework for optimizing energy–data trade-offs in multi-hop WSNs. By leveraging convex optimization and superlinear convergence, our algorithm is deployable on low-power sensor hardware (e.g., ARM Cortex-M series), making it applicable to real-world scenarios such as smart infrastructure, precision agriculture, and remote health monitoring. Future work will focus on extending our system model to environmentally sustainable communication paradigms, such as minimizing source transmit power while guaranteeing minimum QoS requirements, thereby maximizing network lifetime.

## Figures and Tables

**Table 1:** List of the main symbols first introduced in Section 2.

Symbol	Definition
$r_{0}$	The source node
$r_{k}$	The k-th relay node, $k\in \left\{1,\ldots ,K\right\}$
$r_{K+1}$	The destination node
K	The number of the relay nodes, a positive integer, with typical values being 1, 2, 3, 4, and 5
$h_{k}$	The channel coefficient between $r_{k-1}$ and $r_{k}$, $k\in \left\{1,\ldots ,K+1\right\}$
$g_{k}$	The channel gain between $r_{k-1}$ and $r_{k}$, $k\in \left\{1,\ldots ,K+1\right\}$
T	The EH and ID period
$\theta_{k}$	The TS ratio at the k-th relay node, $k\in \left\{1,\ldots ,K\right\}$
θ	$\theta =\left\{\theta_{k}|\theta_{k}\in \left(0,1\right),k=1,\ldots ,K\right\}$
$s_{k}$	The information symbol at $r_{k}$, $k\in \left\{0,\ldots ,K\right\}$
$y_{k}$	The received signal at $r_{k}$, $k\in \left\{1,\ldots ,K+1\right\}$
$p_{k}$	The transmit power at $r_{k}$, $k\in \left\{0,\ldots ,K\right\}$
$n_{k}$	The AWGN at $r_{k}$, $k\in \left\{1,\ldots ,K+1\right\}$
$\delta^{2}$	The variance of the AWGN
$\eta_{k}$	The rectification efficiency of relay node $r_{k}$, $\eta_{k}\in \left(0,1\right]$, $k\in \left\{1,\ldots ,K\right\}$
$e_{k}$	The harvested energy at relay node $r_{k}$, $k\in \left\{1,\ldots ,K\right\}$
W	The transmission bandwidth
$\gamma_{k}$	The received SNR at $r_{k}$, $k\in \left\{1,\ldots ,K+1\right\}$
$A_{1}$	$A_{1}=\frac{g_{1}}{W\delta^{2}}$ (auxiliary constant)
$A_{k}$	$A_{k}=\frac{\prod_{i=1}^{k}g_{i}\prod_{i=1}^{k-1}\eta_{i}}{{W\delta }^{2}}$, $k\in \left\{2,\ldots ,K+1\right\}$ (auxiliary constant)
$R_{k}$	The achievable data rate of hop k, $k\in \left\{1,\ldots ,K+1\right\}$
R	The end-to-end achievable data rate from $r_{0}$ to $r_{K+1}$
$p_{min}$	The minimum transmit power of $r_{0}$
$p_{max}$	The maximum transmit power of $r_{0}$

**Table 2:** List of the main symbols first introduced in Section 3.

Symbol	Definition
$p_{0}^{a}$, $p_{0}^{b}$	Two arbitrary values of $p_{0}$, $p_{0}^{a}<p_{0}^{b}$
$B_{k}$	${B_{k}=A}_{k}p_{max}$, $k\in \left\{1,\ldots ,K+1\right\}$ (auxiliary constant)
$H\left(R_{2}\right)$	The Hessian matrix of $R_{2}$
r	$r=\frac{\min\left\{R_{1},\ldots R_{K+1}\right\}}{W}$ (auxiliary auxiliary)
$x_{k}$	$x_{k}=\ln\theta_{k}$, $k\in \left\{1,\ldots ,K\right\}$ (auxiliary auxiliary)
x	$x=\left\{x_{k}|x_{k}=\ln\theta_{k}\in \left(-\infty ,0\right),k=1,\ldots ,K\right\}$
y	y=lnr (auxiliary auxiliary)
$f_{1}\left(x_{1},y\right)$	$f_{1}\left(x_{1},y\right)=y-\ln\left(1-e^{x_{1}}\right)-\ln\left(\ln\left(1+B_{1}\right)\right)$ (auxiliary function)
$f_{k}\left(x_{1},\ldots ,x_{k},y\right)$	$f_{k}\left(x_{1},\ldots ,x_{k},y\right)=y-\ln\left(1-e^{x_{k}}\right)-\ln\left(\ln\left(1+B_{k}e^{\sum_{i=1}^{k-1}x_{i}}\right)\right)$, $k\in \left\{2,\ldots ,K\right\}$ (auxiliary function)
$f_{K+1}\left(x_{1},\ldots ,x_{K},y\right)$	$f_{K+1}\left(x_{1},\ldots ,x_{K},y\right)=y-\ln\left(\ln\left(1+B_{K+1}e^{\sum_{i=1}^{K}x_{i}}\right)\right)$ (auxiliary function)
$u\left(z\right)$	$u\left(z\right)=-\ln\left(1-e^{z}\right)$ (auxiliary function)
$v_{k}\left(z\right)$	$v_{k}\left(z\right)=-\ln\left(\ln\left(1+B_{k}e^{z}\right)\right)$, $k\in \left\{2,\ldots ,K+1\right\}$ (auxiliary function)
$w_{k-1}\left(x_{1},\ldots ,x_{k-1}\right)$	$w_{k-1}\left(x_{1},\ldots ,x_{k-1}\right)=\sum_{i=1}^{k-1}x_{i}$ (auxiliary function)

**Table 3:** Comparison of several mainstream methods for solving Problem (16).

Method	Initial Point	Performance Characteristics
Interior point method	Feasible point	Convergence rate: Superlinear Computational complexity/iteration: $O\left(N^{3}\right)$
Exterior point method	Arbitrary point	Convergence rate: Sublinear/linear Computational complexity/iteration: $O\left(N^{3}\right)$
Lagrangian dual method	Arbitrary point	Convergence rate: Sublinear Computational complexity/iteration: $O\left(N^{3}\right)$

N is the number of independent variables in Problem (16), N=K+1.

**Table 4:** List of the main symbols first introduced in Section 4 and Section 5.

Symbol	Definition
N	The number of independent variables in Problem (16), N=K+1
$g_{k}\left(x_{k}\right)$	$g_{k}\left(x_{k}\right)=x_{k}$, $k\in \left\{1,\ldots ,K\right\}$ (auxiliary constant)
$\phi \left(x,y\right)$	The log barrier function, $\phi \left(x,y\right)=-\sum_{k=1}^{K+1}\ln\left(-f_{k}\right)-\sum_{k=1}^{K}\ln\left(-g_{k}\right)$
t	The barrier parameter, t>0
$\psi \left(x,y;t\right)$	The barrier objective function, $\psi \left(x,y;t\right)=-y+\frac{1}{t}\phi \left(x,y\right)$
μ	The update factor for t in the outer loop, μ>1
$\epsilon_{o}$	The tolerance for the stopping criterion in the outer loop of the log barrier method, $\epsilon_{o}>0$
$\epsilon_{i}$	The tolerance for the stopping criterion in the inner loop of the log barrier method, $\epsilon_{i}>0$
m	The total number of the constraints in Problem (16), m=2K+1
∇ψ	The gradient of ψ
$\nabla^{2}\psi$	The Hessian matrix of ψ
∆x	The step size of x, $∆x=\left[∆x_{1},\ldots ,∆x_{K}\right]$
∆y	The step size of y
λ	The Newton decrement, $\lambda =\sqrt{-{\nabla \psi }^{T}{\left[∆x,∆y\right]}^{T}}$
α	The step size to update the x and y in the inner loop
β	The constant to update α, $\beta \in \left(0,1\right)$
c	The constant used in the Armijo condition, $c\in \left(0,1\right)$

## Data Availability

The data presented in this study are available upon request from the corresponding author.

## Abbreviations

The following abbreviations are used in this manuscript:

SWIPT	Simultaneous wireless information and power transfer
DF	Decode-and-forward
TS	Time-switching
WSN	Wireless sensor network
IoT	Internet of Things
LoS	Line-of-sight
SNR	Signal-to-noise ratio
EH	Energy harvesting
RF	Radio frequency
PS	Power-splitting
ID	Information decoding
AF	Amplify-and-forward
CSI	Channel state information
QoS	Quality of service
IWSN	Industrial wireless sensor network
SCA	Successive convex approximation
AoI	Age of Information
D2D	Device-to-device
SCP	Secrecy connectivity probability
AI	Artificial intelligence
DNN	Deep neural network
CNN	Convolutional neural network
GPU	Graphics processing unit
AWGN	Additive white Gaussian noise

## Appendix A

From (5) and (6), it follows that $\gamma_{k}$, $k\in \left\{1,\ldots ,K+1\right\}$ is directly proportional to $p_{0}$. Since $\ln\left(1+\gamma_{k}\right)$ is an increasing function of $\gamma_{k}$, $R_{k}$ must be a monotonically increasing function of $p_{0}$.

We now examine the monotonicity of the min-function $\min\left\{R_{1},\ldots .R_{K+1}\right\}$. Consider two arbitrary values of $p_{0}$: $p_{0}^{a}$ and $p_{0}^{b}$ with $p_{0}^{a}<p_{0}^{b}$. Due to the monotonic increasing property of each $R_{k}$, $k\in \left\{1,\ldots ,K+1\right\}$, it holds that:

(A1) $$R_{k}\left(p_{0}^{a}\right)<R_{k}\left(p_{0}^{b}\right).$$

Consequently, the min-function satisfies:

(A2) $$\min\left\{R_{1}\left(p_{0}^{a}\right),\ldots ,R_{K+1}\left(p_{0}^{a}\right)\right\}<\min\left\{R_{1}\left(p_{0}^{b}\right),\ldots ,R_{K+1}\left(p_{0}^{b}\right)\right\}.$$

Thus, the system throughput R is a monotonically increasing function of $p_{0}$.

## Appendix B

The necessary condition for the maximization problem (11) to be convex is that the objective function $\min\left\{R_{1},\ldots .R_{K+1}\right\}$ is concave. We first analyze the concavity of the subfunctions $R_{k}$, $k\in \left\{1,\ldots ,K+1\right\}$.

For $R_{1}$ in (12):

Since $W\ln\left(1+B_{1}\right)$ is a constant term, $R_{1}$ is a linear function and thus both convex and concave.

For $R_{k}$, $k\in \left\{2,\ldots ,K+1\right\}$ in (13) and (14):

The multiplicative term $\prod_{i=1}^{k-1}\theta_{i}$ for $k\in \left\{2,\ldots ,K+1\right\}$ is neither convex nor concave. Consequently, $R_{k}$ is not concave. Take $R_{2}$ as an example:

(A3) $$R_{2}=W\left(1-\theta_{2}\right)\ln\left(1+B_{2}\theta_{1}\right).$$

Its Hessian matrix is:

(A4) $$H\left(R_{2}\right)=\left[\begin{array}{cc} \frac{\partial^{2}R_{2}}{\partial \theta_{1}^{2}} & \frac{\partial^{2}R_{2}}{\partial \theta_{1}\partial \theta_{2}}\\ \frac{\partial^{2}R_{2}}{\partial \theta_{2}\partial \theta_{1}} & \frac{\partial^{2}R_{2}}{\partial \theta_{2}^{2}} \end{array}\right]=\left[\begin{array}{cc} -\frac{W\left(1-\theta_{2}\right)B_{2}^{2}}{{\left(1+B_{2}\theta_{1}\right)}^{2}} & -\frac{WB_{2}}{1+B_{2}\theta_{1}}\\ -\frac{WB_{2}}{1+B_{2}\theta_{1}} & 0 \end{array}\right].$$

The determinant of $H\left(R_{2}\right)$ is:

(A5) $$\det\left(H\left(R_{2}\right)\right)=-{\left(\frac{WB_{2}}{1+B_{2}\theta_{1}}\right)}^{2}<0.$$

Hence, $H\left(R_{2}\right)$ is indefinite, proving that $R_{2}$ is neither convex nor concave.

Since there exists at least one non-concave $R_{k}$ (e.g., $R_{2}$), the pointwise minimum function $\min\left\{R_{1},\ldots .R_{K+1}\right\}$ cannot be concave. Therefore, Problem (11) is a non-convex optimization problem [30].

## Appendix C

The objective function of Problem (16), −y, is linear and thus both convex and concave. According to the definition of a convex optimization problem [30], to prove that Problem (16) is convex, it suffices to show that the following functions are convex:

(A6) $$f_{1}\left(x_{1},y\right)=y-\ln\left(1-e^{x_{1}}\right)-\ln\left(\ln\left(1+B_{1}\right)\right),$$

(A7) $$f_{k}\left(x_{1},\ldots ,x_{k},y\right)=y-\ln\left(1-e^{x_{k}}\right)-\ln\left(\ln\left(1+B_{k}e^{\sum_{i=1}^{k-1}x_{i}}\right)\right),\;k\in \left\{2,\ldots ,K\right\},$$

(A8) $$f_{K+1}\left(x_{1},\ldots ,x_{K},y\right)=y-\ln\left(\ln\left(1+B_{K+1}e^{\sum_{i=1}^{K}x_{i}}\right)\right).$$

Define the auxiliary function $u\left(z\right)=-\ln\left(1-e^{z}\right)$. Its second derivative is:

(A9) $$u^{\prime \prime }\left(z\right)=\frac{e^{z}}{{\left(1-e^{z}\right)}^{2}}>0.$$

Thus, $u\left(z\right)$ is strictly convex for z<0. The function $f_{1}\left(x_{1},y\right)$ is the sum of $u\left(x_{1}\right)$ (convex) and the affine function $y-\ln\left(\ln\left(1+B_{1}\right)\right)$. As the sum of convex and affine functions remains convex, $f_{1}\left(x_{1},y\right)$ is convex.

Define the auxiliary function $v_{k}\left(z\right)=-\ln\left(\ln\left(1+B_{k}e^{z}\right)\right)$, $k\in \left\{2,\ldots ,K+1\right\}$. Its second derivative is:

(A10) $$v_{k}^{\prime \prime }\left(z\right)=-\frac{B_{k}e^{z}\left[\ln\left(1+B_{k}e^{z}\right)-B_{k}e^{z}\right]}{{\left[\left(1+B_{k}e^{z}\right)\ln\left(1+B_{k}e^{z}\right)\right]}^{2}}.$$

Note that $\ln\left(1+B_{k}e^{z}\right)-B_{k}e^{z}<0$ for $B_{k}e^{z}>0$. Thus, $v_{k}^{\prime \prime }\left(z\right)>0$, proving $v_{k}\left(z\right)$ is convex.

Then, define the affine function $w_{k-1}\left(x_{1},\ldots ,x_{k-1}\right)=\sum_{i=1}^{k-1}x_{i}$. Since $v_{k}\left(z\right)$ is convex and $w_{k-1}\left(x_{1},\ldots ,x_{k-1}\right)$ is affine, their composition $v_{k}\left(w_{k-1}\left(x_{1},\ldots ,x_{k-1}\right)\right)$ is convex [30]. Therefore, $f_{k}\left(x_{1},\ldots ,x_{k},y\right)=y+u\left(x_{k}\right)+v_{k}\left(w_{k-1}\left(x_{1},\ldots ,x_{k-1}\right)\right)$ is a sum of convex functions and thus convex.

Similarly, $f_{K+1}\left(x_{1},\ldots ,x_{K},y\right)=y+v_{K+1}\left(w_{K}\left(x_{1},\ldots ,x_{K}\right)\right)$ is convex.

In summary, the objective function −y is linear (hence convex), and all inequality constraint functions (A6)–(A8) are convex. The feasible set defined by $x_{k}<0$, $k\in \left\{1,\ldots ,K\right\}$ is a convex set. Therefore, Problem (16) is a convex optimization problem.
